# Diseases outside the Mouth That Affect the Oral Structures

**Published:** 1888-06

**Authors:** J. Y. Martin

**Affiliations:** Ann Arbor, Mich.


ARTICLE IV.

### DISEASES OUTSIDE THE MOUTH THAT AFFECT THE ORAL STRUCTURES.

BY DR. J. Y. MARTIN, ANN ARBOR, MICH.

[Read before the Michigan State Dental Association, held at Ann Arbor, Mich., March 20, 1888.]

The teeth first begin to form in the early part of fœtal life. Beginning about the seventh week, the process gradually goes on until by the eleventh week have the germs for the set of milk teeth. There are variations in some cases, but these are the usual periods. These germs are of elements we little suspect. The hardest portions are composed of soft-cells. They are composed of epithelial cells at first loosely grouped together.

There are certain laws that govern the development of all parts of the body. They are most marked in the fœtal state. As long as Nature's laws are not interfered with, everything goes on smoothly. But if a law is broken we find its influence on the fœtus. It is very sensitive to impressions. If there is an arrest of development at any time, the organ that is then developing will be absent or deformed. Great care should be exercised at this time, since any irregularity may show its effects upon the fœtus and result in various deformities, as cleft palate and harelip.

Maternal impressions during fœtal life may produce these results. They may be on the nervous or physical system. If on the former, we may have various mental and nervous disturbances. If they affect the physical system, the organ in process of development will be absent or imperfectly developed. What are the effects of disease in the mother or in the fœtus? The relation between them is so close that whatever is good for the one is good for the other and whatever is bad for the one is bad for the other. Hence diseases that prey upon the mother during the fœtal life of her offspring may produce lasting impressions upon it. Thus the child may inherit from its mother certain diseased conditions, as a weakened digestion, nervous temperament and other conditions that will interfere with the nutrition and growth of the child.

After birth, as a result of a few weeks's fever, the child's teeth may be destroyed; for example, scarlet fever, small pox, or typhoid fever may so alter nutrition and the secretions as to produce these results. These results are not confined to infant life but may effect the adult as well. The teeth contain organic and inorganic matter. If they are not properly nourished, the organic matter is diminished and the teeth become more friable than is normal, and are not able to withstand external influences.

The subject of dentition is looked upon by many as if all the disturbances in the system at this time were due to the eruption of the teeth. The child is supposed to live upon milk food up to this time. When the teeth are being erupted, there are certain important changes taking place in the intestinal canal, and the disturbances in the mouth are but local manifestations of general disturbances. We have changes in the oral cavities, to be sure, but at the same time, new glands are forming and other changes are taking place along the alimentary canal. The child is being prepared to digest a different kind of food and the digestive organs must be changed accordingly.

What is the condition of the child during this transitional period? Probably nature intended there should be little disturbance in the mouth and system generally; and this would be so were it not that hygienic laws have been violated either before or after birth. Whenever we violate Nature's laws, we must pay the penalty. At this time there are changes not only in the alimentary canal and mouth, but in the nervous system as well, and these changes are in proportion to the amount of interference with nutrition at this period. The nervous system becomes more susceptible, and impressions made on the child are more marked. This accounts for the convulsions and the various nervous disturbances at this time.

The eruption of teeth is a physiological process and in itself should produce little disturbance. But as the child is erupting teeth we are apt to attribute all these dis urbances to this, forgetting that all these changes are taking place at one time, while the eruption of teeth is but a small part of the cause. The tongue is an indicator of general disturbances. But why look at the tongue only? Other changed conditions in the oral cavity are indicators as well. Acid secretions in the mouth are as strong indicators of abnormal conditions in the system as are the changed conditions in the tongue. In nine cases out of ten acid secretions in the mouth mean some change outside of the mouth. Whenever we have tumid gums more often the cause exists outside of the mouth than within. When we have tartar upon the teeth and the gums are tumid, we say the tartar is the cause of the tumid gums, while in reality the presence of the tartar is due to the excess of the carbonate or phosphate of lime in the secretions, and this excess is caused by a changed condition without the mouth.

Now as to saliva. In its normal state it is a bluish white, ropy secretion, very little heavier than water. Normally it is alkaline in reaction. It is never in a marked degree and without some special cause, usually outside the mouth. Normal saliva changes so rapidly that if we wish to examine it, it must be done at once. Abnormal saliva may contain pus, blood, carbonates and phosphates of lime, etc. From the excess of the carbonates and phosphates of lime the tartar is produced and the diseases that come from this accumulation. The offensive character of the tartar is due to the presence of organic matter. Normal saliva of the child is similar to that of the adult, except that alkalinity is more marked. If the saliva become acid, its effects upon the oral structures are more marked.

In the common summer complaints of infants, such as indigestion and diarrhœa, the secretions of the mouth are generally acid. There is an excess of acid in the system, due either to over-production or lack of proper elimination. When there is an excess of acid in the system, due either to indigestion or other causes, it irritates the alimentary canal and the mucous membrane of the mouth. Thus results increased irritation. If the secretions in cholera infantum or diarrhœa be examined, they will be found in a marked degree acid. The food, instead of being digested, undergoes acid fermentation. When acids are in excess they are absorbed into the system. The buccal secretions consequently may become more acid and the saliva rendered neutral or acid in reaction.

Causes of acid secretions in the mouth, according to Wright: 1. Inflammation of the mucous membrane of the mouth. 2. Diseases of the salivary glands. 3. Diseases in which there is an excess of acid in the system, as rheumatism and gout. 4. Indigestion or dyspepsia.

In inflammation of the mouth, we first get under secretions, then over-secretion; excess of buccal and other secretions. Then if the mucous membrane breaks down by ulceration we have pus mingling with the secretions. The acid in excess is due either to excess of buccal secretions, diminished flow of saliva or changed saliva.

Catarrh has a marked effect on the glands of the mucous membrane wherever it occurs. It causes excessive secretion and later the secretions may become abnormal from over-work of the glands. This secretion may pass into the stomach and mingle with the digestive secretions and alter their condition.

Acid secretions favor the development of certain parasites, which cause one of the worst forms of ulcers in the mouth. Thus the result of another cause acts in producing secondary diseases in the oral cavity.

Another cause of acid secretions is disease of tbe salivary glands. They may be affected so as to cause diminution in the action of the glands and either diminish the alkalinity or change reaction to acid. One salivary gland may be diseased and secrete abnormally, the other may be normal; then we may have acid secretions.

In rheumatism and gout, there is an over-production of acid in the system. Over-production of acid in the system may end in change of salivary secretion, so that instead of being alkaline it becomes acid. It has been tested and found to be as stated. We notice this change in the mouth. We do not always know its cases. It is often attributed to disease of the liver. It is claimed that when there is an excess of acid secretions in the system it is generally due to a lack of proper elimination of effete matter from the system.

*Indigestion or dyspepsia.*—It may be either atonic or variety. The former is due to lack of force in the digestive organs; the latter, to inflammation or congestion. What is the result? Food passes into the stomach at the temperature of 98½°, and if not properly digested undergoes changes that will increase the derangements. If the secretions and physiological actions are normal, the food is digested; if not, a long train of ills results. Whatever the cause may be, the results are about the same: we have imperfect digestion and as a result acid and gaseous fermentation. As a result of this gaseous distention of the stomach, food is not properly mingled with the secretions, and this increases the difficulty by retarding still more the digestive process. Also by the distended condition of the stomach and bowels, increased pressure upon the diaphragm results, the chest cavity becomes narrowed; the great venous vessels from the head are pressed upon. Blood will be pumped into the head since the arteries are not so compressed, and, as it cannot return, we have permanent congestion of the head, face and oral structures, and from these may result changes in the oral structures. Indigestion or dyspepsia exists but a short time before the secretions in the mouth become changed, and in many cases in a marked degree, acid. Some claim that as the teeth are developed from the epithelial structures they are affected the more by the change in the secretions.

During gestation there is frequently a marked disturbance of the digestive organs: indigestion, marked nausea, vomiting and sometimes total arrest of digestive process. The secretions of the mouth in this condition become changed in a marked degree, and in some cases muriatic acid is found in them in addition to other acids, even when vomiting does not take place. It has been noticed for years that during gestation the oral structures and especially the teeth suffer greally. One author said, many years ago, "A tooth for a child." More recent authors claim that the destructive process is often still greater than that during the period of gestation, all of which is due to changed nutrition and altered secretions, which in turn are due to indigestion or dyspepsia.

The effects of acid secretions upon the teeth are too well known to you as dentists for me to speak of them in detail. What interests you most is, Where and what are the causes? Nine times out of ten the causes are without the mouth. To point out some of these has been my purpose in this brief talk.

From our present standpoint, it is easy to see how ineffectual alkaline mouth-washes are for the relief of a patient who is suffering from acid secretions in the mouth which are destroying the teeth. It would be like pinching the tip of the tail of a snake to kill the snake.

Not unfrequently the dentist will say: "I have done the mechanical work to the best of my ability, yet I find that the fillings will not stand." All dentists have that experience. When the work is done well and the filling will not stand, or the teeth decay rapidly, the cause is not, as a rule, in the mouth; but the trouble is due to changed recretions in the mouth or lack of proper nutrition. The teeth must be properly nourished; if not, they become more vulnerable, are more apt to decay, and decay more rapidly when once started.

*As to treatment.*—To treat the patient scientifically, we must treat each disease on its own merits. It is not expected that the dentist will treat all the diseases that produce changes in the mouth, but it is his duty to be able to recognize the trouble and advise the patients to have treatment. By so doing, he confers a boon upon the patient and at the same time protects his own professional skill or reputation.—*Ohio Dental Journal.*

